# Effect of Ethanol and Urea as Solvent Additives on PSS–PDADMA Polyelectrolyte Complexation

**DOI:** 10.1021/acs.macromol.1c02533

**Published:** 2022-04-15

**Authors:** Mohammad Khavani, Piotr Batys, Suvesh M. Lalwani, Chikaodinaka I. Eneh, Anna Leino, Jodie L. Lutkenhaus, Maria Sammalkorpi

**Affiliations:** † Department of Chemistry and Materials Science, School of Chemical Engineering, Aalto University, P.O. Box 16100, FI-00076 Aalto, Finland; ‡ Jerzy Haber Institute of Catalysis and Surface Chemistry, Polish Academy of Sciences, Niezapominajek 8, PL-30239 Krakow, Poland; ^§^Artie McFerrin Department of Chemical Engineering and ^∥^Department of Materials Science and Engineering, Texas A&M University, College Station, Texas 77843, United States; ⊥ Department of Bioproducts and Biosystems, School of Chemical Engineering, Aalto University, P.O. Box 16100, FI-00076 Aalto, Finland; # Academy of Finland Centre of Excellence in Life-Inspired Hybrid Materials (LIBER), Aalto University, P.O. Box 16100, FI-00076 Aalto, Finland

## Abstract

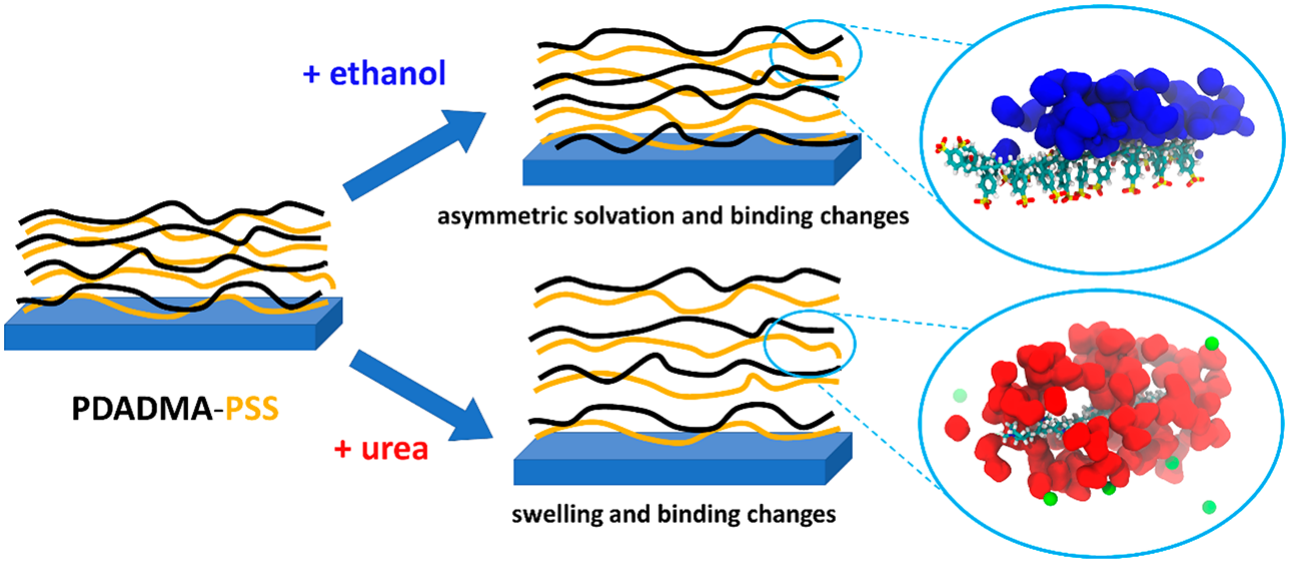

The effect of urea and ethanol additives on aqueous solutions of poly(styrenesulfonate) (PSS), poly(diallyldimethylammonium) (PDADMA), and their complexation interactions are examined here via molecular dynamics simulations, interconnected laser Doppler velocimetry, and quartz crystal microbalance with dissipation. It is found that urea and ethanol have significant, yet opposite influences on PSS and PDADMA solvation and interactions. Notably, ethanol is systematically depleted from solvating the charge groups but condenses at the hydrophobic backbone of PSS. As a consequence of the poorer solvation environment for the ionic groups, ethanol significantly increases the extent of counterion condensation. On the other hand, urea readily solvates both polyelectrolytes and replaces water in solvation. For PSS, urea causes disruption of the hydrogen bonding of the PSS headgroup with water. In PSS–PDADMA complexation, these differences influence changes in the binding configurations relative to the case of pure water. Specifically, added ethanol leads to loosening of the complex caused by the enhancement of counterion condensation; added urea pushes polyelectrolyte chains further apart because of the formation of a persistent solvation shell. In total, we find that the effects of urea and ethanol rise from changes in the microscopic-level solvation environment and conformation resulting from solvating water being replaced by the additive. The differences cannot be explained purely via considering relative permittivity and continuum level electrostatic screening. Taken together, the findings could bear significance in tuning polyelectrolyte materials’ mechanical and swelling characteristics via solution additives.

## Introduction

Polyelectrolytes (PEs) are macromolecules that dissociate in aqueous solutions into charged macromolecules and their counterions. Oppositely charged PEs can spontaneously associate in these solutions to form polyelectrolyte complexes (PECs) or structurally related polyelectrolyte multilayers (PEMs).^1^ PE materials have raised significant interest during the past decades because of their tunable, versatile properties^2^ and scalable processing techniques for industrial applications in pharmaceutical sciences and biomedicine, energy materials, and responsive, functional films and assemblies.^2−4^

PE complexation is propelled by the interplay of the PE–PE ion pair formation and the related counterion release entropy.^5^ Other intermolecular interactions, including hydrogen bonding, van der Waals forces, hydrophobic interactions, and dipole interactions, also influence complex formation.^3^ Polymer characteristics, such as charge density, molecular weight, chain flexibility, chirality, and polymer structure, further influence the formed assemblies.^6−10^ Solution or assembly conditions also play a role; PE assemblies are strongly sensitive to salt and added ions,^6,11,12^ hydration,^13,14^ and other solvation conditions, such as temperature^15^ and pH.^13,16^ Recent work highlights the additional role of the water binding ability at the PE ion pairs;^15,17−19^ i.e., local solvent conditions are important.

The importance of water binding and hydration conditions indicates also that PE materials properties can be expected to depend on solvent composition. At macroscale, the relative permittivity of the solvent influences PE assembly. At microscale, the hydrogen bond network of bulk water contributes significantly to a PE’s solvation characteristics. Consequently, a solvent with different hydrogen-bonding ability influences both PE solvation and assembly characteristics. For example, ethanol as a more hydrophobic, lower relative permittivity solvent leads to poorer solvation of PEs than water.^20^ As another consideration, solvent additives influence the water hydrogen bond network, thus affecting the response of PEs. For example, urea, which is a hydrogen bond breaker, has a drastic effect on PE hydrogel swelling.^21,22^

Studies on the effect of solvent composition on PE materials have shown that, for example, the growth and structure of PEMs of both poly(styrenesulfonate) (PSS) and poly(allylamine hydrochloride) (PAH)^23^ and PSS and poly(diallyldimethylammonium) (PDADMA)^24,25^ can be controlled by changing the amount of ethanol in the PE assembly. Decreasing the solvent quality, i.e., increasing the amount of ethanol in assembly solution, leads to an increase in film thickness and mass. In line with these studies and pointing toward swelling, PSS/PDADMA capsule permeability increases in the presence of ethanol.^26^ However, PEMs involving hyaluronic acid (HA)/chitosan and PSS/PDADMA exhibit wide-ranging and PE-dependent swelling properties in ethanol and permeability that does not correlate with ethanol uptake.^27^ Recently, Meng et al.^28^ studied the impact of cosolvent (ethanol/water) on the solid-to-liquid and liquid-to-solution phase transition in polyelectrolyte complexes (PECs). The authors were able to systematically lower the salt concentration required for the phase transition and disassembly by selecting different ratios of ethanol/water. Altogether, the findings point toward a high chemistry specificity in solvation dependency on the ethanol additive. Furthermore, competing influence of the solvent additive on PE ion pairing and PEM hydrophobicity could contribute to the findings.^27^ Indeed, ethanol and ethylene glycol influence the salt response of poly(vinylbenzyltrimethylammonium chloride) (PVBTMA) and PSS complexes.^28^ Alcohol additives also suppress the thermal plasticization transition that hydrated PE assemblies undergo.^19^ Other solution additives besides alcohols have been studied much less. In one example, added urea makes the swelling response of weak PE gels under pH changes stronger.^21,22^

Motivated by this past work, we focus here on resolving the influence of solvent additives on PE solvation and their associative interactions via molecular dynamics (MD) simulations, laser Doppler velocimetry, and quartz crystal microbalance characterization. We focus upon the well-studied PSS/PDADMA PE system and ethanol and urea solvent additives. This polyelectrolyte pairing has been thoroughly studied both by us^6,11,13,17−19,29,30^ and others^32−38^ in water solutions, making it a well-characterized system, both experimentally and theoretically. In addition, the effects of salt on PSS/PDADMA assemblies have been examined significantly.^6,11,17,38−42^ Also, the dynamics of water in PECs has recently raised significant interest.^43^ However, these prior studies largely did not consider the effects of solvent additives such as ethanol or urea rising from the microscopic or molecular solvation level. Ethanol is chosen here as a practically significant, common solvent additive that exhibits a permittivity lower than that of water. For comparison, urea is chosen here as another common additive that exhibits hydrogen bond breaking character. The results of this work are discussed in the context of each solvent additive’s influence on the PEs and their interactions, thus connecting the findings on macroscopic film structure to an enhanced control of PE materials properties.

## Materials and Methods

### Materials

Poly(diallyldimethylammonium chloride) (PDADMA, *M*_w_ = 200000–350000 g/mol, 20 wt % solution), poly(styrenesulfonate sodium salt) (PSS, *M*_w_ = 500000 g/mol), and linear polyethylenimine (LPEI, *M*_w_ = 25000 g/mol) were purchased from Sigma-Aldrich, Scientific Polymer Products, and Polysciences, Inc., respectively. Sodium chloride (NaCl), 4-cyano-4-(phenylcarbonothioylthio)pentanoic acid (CPhPA), pure ethanol (≥99.5% ACS reagent), and urea were purchased from Sigma-Aldrich. Sodium 4-styrenesulfonate (SSNa) (98% HPLC) was purchased from AK Scientific. 4,4′-Azobis(4-cyanovaleric acid) (V-501) was purchased from FUJIFILM Wako Pure Chemical Corporation. Silicon dioxide-coated QSensors (QSX 303 SiO_2_) were used as substrates and were purchased from Biolin Scientific. Dialysis tubing with a molecular weight cutoff (MWCO) of 3.5 kDa was purchased from VWR. Milli-Q water with a resistivity of 18.2 MΩ·cm was used for all experiments.

### Polymer Synthesis

Poly(sodium 4-styrenesulfonate) (PSS) used for laser Doppler velocimetry (LDV) tests was synthesized via aqueous reversible addition–fragmentation chain transfer (RAFT) polymerization. A 15 mL aqueous solution containing SSNa monomer (24.3 mmol, 5.00 g), CPhPA RAFT agent (0.196 mmol, 54.8 mg), and V-501 initiator (0.059 mmol, 16.5 mg) was prepared in a 50 mL round-bottom flask. A molar equivalence of 124:0.3:1 of monomer to initiator to RAFT agent was maintained. The flask was sealed, the solution was stirred, and nitrogen was bubbled through the solution for 30 min. Then, the round-bottom flask was placed in an oil bath maintained at 70 °C for 5 h. The reaction was stopped by exposing the reaction solution to air and cooling it to room temperature by using ice-cold water. Next, the reaction solution was dialyzed against Milli-Q water for 48 h. The dialyzing water was changed once every 12 h. Finally, the polymer was recovered by lyophilization and drying at 50 °C.

### Polymer Characterization

After synthesis, the polymer was characterized by using proton nuclear magnetic resonance (^1^H NMR) spectroscopy, size exclusion chromatography (SEC), and modulated differential scanning calorimetry (MDSC). For ^1^H NMR spectroscopy, 10 mg of polymer was dissolved in 1 mL of D_2_O, and a 400 MHz Bruker NMR was used to collect the ^1^H NMR spectra. The NMR spectra are shown in Figure S1. MDSC was performed by using a TA Q200 differential scanning calorimeter (Figure S2). A procedure developed by Shao et al. was used to measure the glass transition temperature of dry polyelectrolyte.^44^ SEC was performed on a TOSOH EcoSEC with UV (254 nm) and RI detectors at 25 °C. The mobile phase was a mixture of 80 vol % 0.3 M NaNO_3_ + 0.01 M NaH_2_PO_4_ at pH 9 + 20 vol % CH_3_OH with a flow rate of 1.0 mL/min. The molecular weight was calculated by using a calibration curve based on poly(ethylene oxide) standards (Figure S3).

### Laser Doppler Velocimetry (LDV)

Zeta-potential (ζ) measurements of polymer solutions were performed by using a dynamic light scattering instrument (Zetasizer, Malvern Instruments) and the appropriate capillary cell, DTS 1070, from Malvern Instruments. The ζ measurements for PDADMA and PSS were performed at a concentration of 0.6 and 0.1 mg/mL, respectively, in water, 10 and 30 wt % ethanol, and urea solutions. The ζ measurements for PSS were performed by using the synthesized PSS. All zeta-potential measurements were performed after filtering the solutions through 0.45 μm PTFE syringe filter.

### Quartz Crystal Microbalance with Dissipation (QCM-D) Monitoring

Layer-by-layer (LbL) assembly of five layer pairs of PDADMA/PSS polyelectrolyte multilayers films prepared with an LPEI anchor layer (LPEI(PSS/PDADMA)_5_) was monitored by using the QSense E4 instrument. LbL films were assembled on SiO_2_ AT-cut quartz crystals with a resonant frequency of 4.95 MHz. Each new crystal was cleaned by plasma treatment using an O_2_-plasma etcher for 15 min, rinsed with Milli-Q water, and dried with compressed air. All QCM-D experiments were performed in triplicate at a set temperature of 23 °C. PDADMA and PSS polyelectrolyte solutions were prepared at a concentration of 0.1 g/L at 0.5 M NaCl. Rinse solutions were prepared at a matching concentration of 0.5 M NaCl. The 0.1 g/L LPEI solution was adjusted to pH 5.5 to obtain a stable solution. All polyelectrolyte and rinse solutions were flowed through a peristaltic pump at a constant flow rate of 150 μL/min. Milli-Q water (pH 5.5) was first allowed to flow over the quartz crystal for 20 min as a baseline for each measurement. An anchor layer of LPEI (pH 5.5) was then deposited onto the crystal for 10 min before rinsing for 5 min with 0.5 M NaCl. Five layer pairs of LPEI(PSS/PDADMA)_5_ were then prepared by an alternating deposition of PSS and PDADMA solutions for 10 min each separated by a 5 min rinse. The assembled films were then exposed to solutions of varying concentrations of ethanol or urea in the presence of 0.5 M NaCl. QTools modeling software (Biolin Scientific) was used to analyze the changes in frequency and dissipation to determine the film thickness. The extended viscoelastic model was used to fit the third, fifth, seventh, and ninth overtones. The L1 film density was set at 1050 g/L and the fluid density set at 1050 g/L for LbL assembly, 970–990 g/L for ethanol, and 1010–1070 g/L for urea.

### Profilometry

After QCM-D experiments, the films were dried under ambient conditions for 24 h and then dried under vacuum at 115 °C for 3 h. The dried film thickness was then measured by using a profilometer (KLA Tencor D-500).

### Molecular Dynamics Simulations

**Figure 1:**
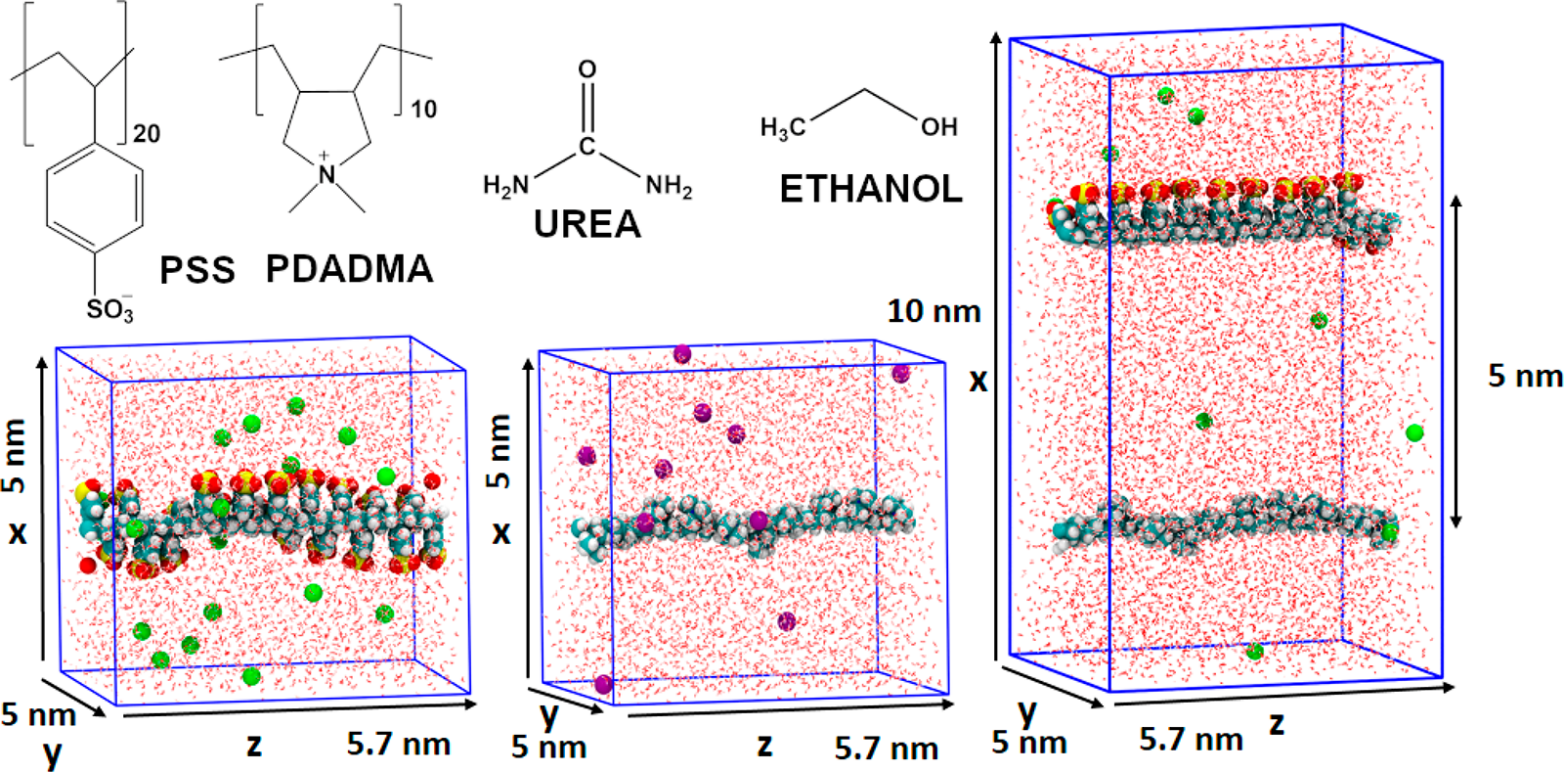
Chemical structure of PSS, PDADMA, ethanol, and urea and the initial simulation configurations corresponding to the single PE chains and the PSS–PDADMA complex. The simulation box dimensions are presented. The PSS and PDADMA are 20 and 10 monomers in length, respectively, and span the simulation box *z*-axially as periodic, infinite chains. The sodium counterions are presented in green and the chloride ions in purple.

All-atom molecular dynamics simulations of PSS and PDADMA in water solution and with ethanol and urea as solvent additives were performed with the Gromacs 5.1.4 package.^45−47^ The PSS and PDADMA chains and ions were described with the OPLS-AA force field^48^ and the ammonium groups extension.^49^ For the partial charges of PSS, parameters originating from ref (36) were used. Sodium and chloride ion models are those of refs (50) and (51), respectively. For water, in compliance with the force-field choice, the explicit TIP4P water model^52^ was employed. PSS and PDADMA chains of 20 and 10 monomers in length, respectively, were examined in simulations of single PEs or simulations encompassing both a PSS and a PDADMA chain, both in the trans configuration. For the single PE simulations, the PE chains were set into the MD simulations box so that they spanned the Cartesian box as straight, *z*-axial chains connected across the periodic boundary conditions to form an infinite chain (see Figure 1). The preparation of the initial configurations followed the protocol presented in ref (53). By use of this protocol, both the PSS and PDADMA chains correspond to extended chain lengths of 5.7 nm. The single chain simulation box size was 5 nm × 5 nm × 5.7 nm. For the simulations with two PEs, the simulation configurations of the single PSS and PDADMA chains were placed in a same simulation box, at initial axial distance of 5 nm (see Figure 1). Two repeat runs of the two-PE complex system were performed, differing by the PSS chain being 90° rotated around its axis to reduce any bias resulting from the initial orientation of the charged groups in the configurations. For the two-PE simulations, a simulation box of size 10 nm × 5 nm × 5.7 nm was used. Radial distribution function *g*(*r*) analyses where the backbone atoms are used as the reference are calculated in 2D in the *xy*-plane taking the *z*-axis as the reference. When atoms (S and N) are used as a reference, the *g*(*r*) calculation is standard 3D.

To investigate the effect of urea and ethanol additives on the PEs and their complexation, both the single PE and the polycation–polyanion chain systems solvated with water, water–ethanol mixtures with either 10 or 30 wt % ethanol, and water–urea mixture with 10 or 30 wt % urea were examined. In each system, enough Na^+^ and Cl^–^ counterions to neutralize the system were added.

After initial energy minimization by the steepest descent method, the single chains and the two-PE chain complex were equilibrated by 100 and 200 ns *NPT* MD simulations, respectively. The production runs used for data analysis were another 100 and 300 ns, respectively. The *NPT* simulations employed the V-rescale thermostat^54^ with a coupling constant of 0.1 ps and a reference temperature *T* = 300 K. The pressure was controlled via the semi-isotropic Parrinello–Rahman barostat^55^ with a coupling constant of 1 ps and a reference pressure of 1 bar. The semi-isotropic barostat was set so that changes of the *x* and *y* dimensions of the box accounted for the pressure control while the *z* axial compressibility was set to zero. The long-range electrostatic interactions were calculated by using the PME method^56^ while the van der Waals interactions were described by using the Lennard-Jones potential and a 1.0 nm cutoff (direct cutoff, no shift). LINCS^57^ and SETTLE^58^ algorithms constrained the bonds in the PEs and water molecules, respectively. A 2 fs time step was used for integrating the equations of motion. For visualizations, VMD has been used.^59^

## Results

### PE–Counterion Interactions

**Figure 2:**
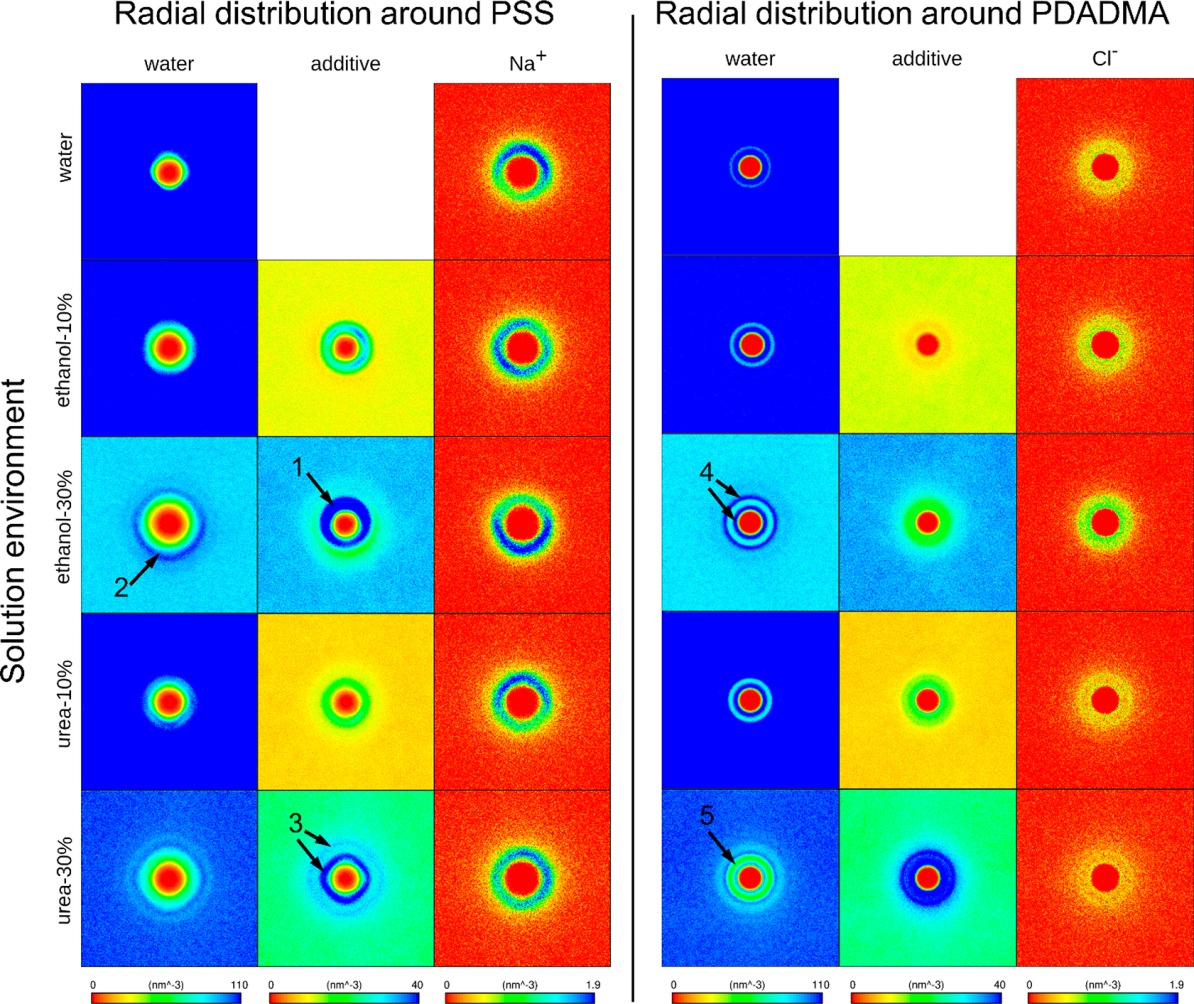
Density maps of the solvent components, i.e., water, ethanol, or urea, and the Na^+^ or Cl^–^ counterions around PSS and PDADMA. In the analysis, the PE is centered with respect to backbone center of mass in all frames, and the presented data are average over 100 ns. The color bars indicate the number density scale of each column. The meanings of the arrows 1–5 are explained in the main text.

In aqueous solution, both PSS and PDADMA are readily soluble, and their counterions dissociate in the solution. We first analyzed the effect of the ethanol and urea solvent additives on the counterion and water distribution around single PE chains in solution. The response is summarized in Figure 2 by 2D density maps of the solvent components and the counterions around PSS and PDADMA. Figures S4 and S5 provide radial distribution function (RDF) based analysis of the water and ion distributions, respectively. Figure S6 presents the ion distribution RDF time dependency, demonstrating convergence of the configurations. Figures S7 and S8 show the corresponding 1D density graphs.

The 2D density maps in Figure 2 show distinct differences between solvent structure around each PE, which we attribute to differences in the chemistries of PSS and PDADMA. The nonuniform distribution of Na^+^ ions (see Figure 2) around PSS chains for all solvent compositions suggests that the charged sulfonate groups extend into solution on one side of the PE chain, leaving a hydrophobic backbone on the other side. In contrast, the distribution of Cl^–^ ions was more evenly spread out spherically around the PDADMA axis (see Figure 2) for all solvent compositions.

With regard to PSS, ethanol has a strong preference for the hydrophobic backbone, pushing water away from that locale, as indicated by arrow 1 in Figure 2. This effect is clearly visible already at 10 wt % ethanol but even more pronounced for 30 wt %. In contrast, a pronounced depletion circle of ethanol persists near the charged sulfonate groups. In this depletion circle, the ethanol density is lower, but the water density higher (see arrow 2 in Figure 2).

Urea, on the other hand, orients strongly around PSS, as indicated by the concentric condensation circles around the PSS axis (Figure 2). The orientation of urea is not guided by the hydrophobic–hydrophilic differences of the PSS chain but more by the change in solution environment in comparison to water. As expected, the water density shows depletion in regions corresponding to the condensed urea pushing away water. The layering of urea is significant, and its concentration becomes ∼100% enriched in comparison to bulk concentration in the condensation stripes (see arrow 3 in Figure 2).

In the 2D density graphs of PDADMA in Figure 2, the most evident feature is the concentric circles in the water density maps (see arrow 4). This indicates strong radial correlation of water around the PDADMA axis which results from the axially symmetric, sterically methyl group screened distribution of PDADMA charge in comparison to more solvent accessible PSS charge. Similar as to around PSS, ethanol depletes systematically from around the PDADMA chain in comparison to bulk ethanol concentration, as demonstrated by the deviation from the background color, but here this depletion is also associated with enhancement of the water layering, i.e., more clearly visible condensation circles of water. Urea, on the other hand, forms a relatively wide, even condensation layer around PDADMA, again significantly enriched in concentration in comparison to bulk solution. This urea condensation layer also partially disrupts the water layering, especially at 30 wt % urea, pushing away the small water molecules that could by their orientation induce the condensation circles (see arrow 5 in Figure 2).

Taken together, the data presented in Figure 2 signify that both the PSS and PDADMA chains experience a significantly different solution environment in the presence of ethanol and urea in comparison to water. It is interesting to consider the counterion condensation response to these changes. As shown by the counterion condensation graphs of Figure 2, counterion condensation strongly follows the charged groups of PSS; i.e., the ions reside at one side of the PE. Interestingly, added ethanol enhances this counterion condensation. The same effect is observed for PDADMA, i.e., stronger counterion condensation with added ethanol. These effects are attributed to the depletion of ethanol around the PEs, which results in a poorer solvent environment for the ionic groups and ions as compared to pure water. Because there is proportionally less ethanol and more water close to the PEs, the small counterions condense as a result of the better solvation environment for them. Urea, on the other hand, condenses strongly around both PEs, which results in it pushing away the counterions due to their preference for aqueous solvation. The diffuse counterion cloud around PDADMA in urea solutions is one such example of this behavior (Figure 2b). The radial distributions of water and ions around each PE (Figures S4 and S5) quantify the differences discussed above.

**Figure 3:**
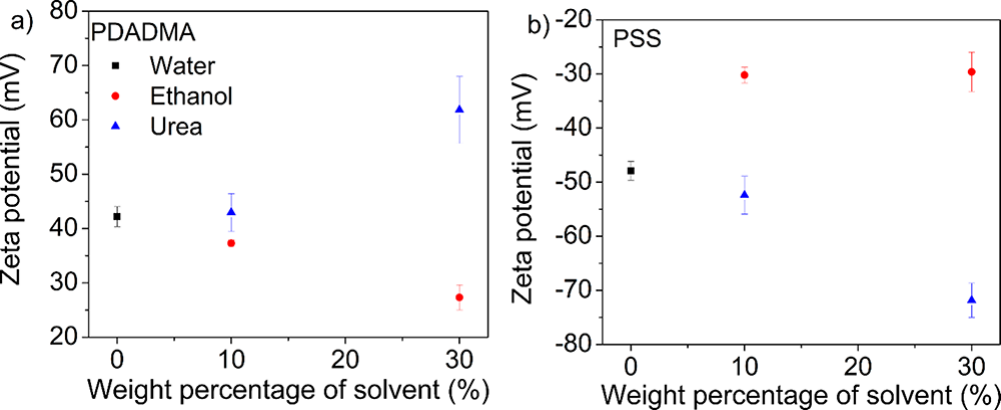
(a) Zeta-potential for PDADMA in water (black squares) and 10 or 30 wt % ethanol (red circles) and urea (blue triangles) solutions. (b) Zeta-potential for PSS in water and 10 or 30 wt % ethanol and urea solutions. The legend in panel (a) applies to (b).

To complement the findings from MD simulations, zeta-potential (ζ) measurements were performed to study the effect of solvent on counterion condensation. Figure 3 shows ζ for PDADMA and PSS in 10 and 30 wt % ethanol and urea solutions. For both PDADMA and PSS, the addition of ethanol reduced the absolute value of ζ when compared against pure water. The decrease in the absolute value of ζ was less pronounced for PSS when going from 10 to 30 wt % ethanol solution. In contrast, addition of urea increased the absolute value of ζ when compared against pure water. For both PSS and PDADMA, a pronounced increase in the absolute value of ζ was observed when going from 10 to 30 wt % urea solution. These results complement the MD simulations in which counterions condensed in the presence of ethanol manifest as a decrease in the experimentally measured absolute value of ζ. As for urea, MD simulations show that urea pushes away the counterions, which manifests in an increase in the experimental absolute value of ζ.

**Figure 4:**
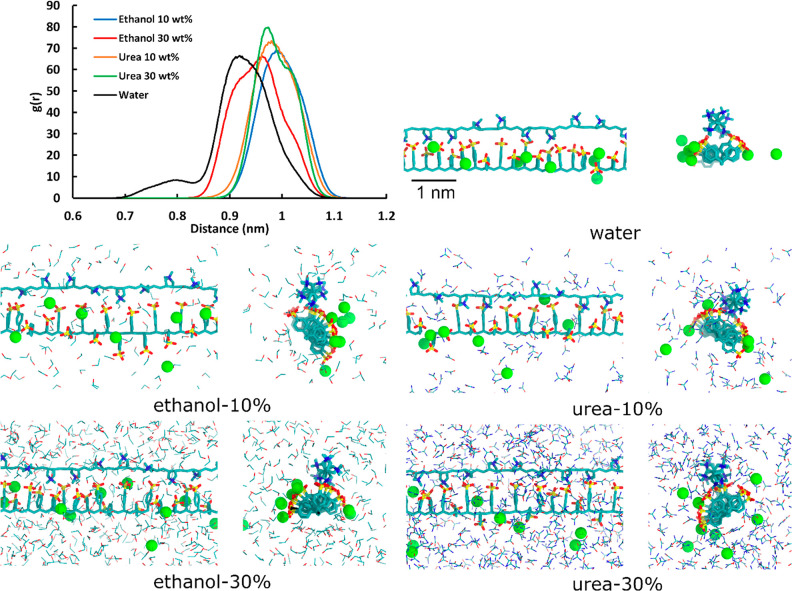
2D radial distribution functions *g*(*r*) calculated between the backbone atoms of the two PEs in different solution environments and visualizations of PSS–PDADMA complexes formed in water and with the solvent additives. Both side and axial views are presented. The green spheres represent the Na^+^ ions neutralizing the complex. Water molecules were omitted for clarity. The *g*(*r*) analysis was done for the 200–500 ns time period of the MD simulations, and the visualizations were selected from the same time period as representative of the binding configurations.

Let us next consider the influence of ethanol and urea solvent additives on PSS–PDADMA complexation. Figure 4 presents characterization of the PSS–PDADMA complexes in terms of complexation structure and PE–PE binding. The presented radial distribution function *g*(*r*) data show that the solvent additives influence the PSS–PDADMA distance in the PE complex. For the complex in water, two distinct binding peaks are visible in radial distribution function of the backbone atoms: one around 0.8 nm and another around 0.9 nm (Figure 4). These correspond to two distinct complexation configurations. The configuration corresponding to the 0.8 nm peak is presented in Figure 4 as the visualization of the PE complex in water, and the 0.9 nm binding configuration is presented as the visualization of the PE complex in the ethanol and urea additives (Figure 4). In the 0.8 nm backbone separation configuration, the PDADMA chain resides between the PSS side chains that alternate at different sides around it, giving rise to a rather tightly bound complex with nearly all PSS charge groups in contact with PDADMA charge groups. Notably, PSS monomers correspond to a shorter backbone than PDADMA monomers which leads to PSS line charge density exceeding PDADMA line charge density significantly: this configuration arises because of the alternating symmetry of the sulfonate group positioning. The degree of intrinsic charge compensation is very high. On the other hand, the 0.9 nm backbone separation configuration was present in all examined solvent compositions; approximately half of the PSS charge groups point toward the PDADMA chain charges. The remainder of the PSS charge groups point away from the PDADMA chain toward the solution due to steric considerations. This leads to an intrinsic compensation ratio that matches the line charge densities of the PEs, with PDADMA charge compensated intrinsically to a very high degree. This configuration is visualized for e.g. ethanol at 10 wt %, but also other solvent additive visualizations show different dynamic variations.

The effects of the additive are next discussed. Introducing 10 wt % ethanol results in the system no longer exhibiting the shorter backbone separation configuration of PE complexes in water. No drastic changes in binding configuration occur going from 10 to 30 wt % ethanol, but 30 wt % ethanol moves the PEs further apart. The distance change is comparatively small and likely results from an excess of condensing counterions providing charge screening for the PSS charge groups. The decreasing electrostatic attraction moves the PDADMA chain slightly further away from the PSS chain; however, the influence is not sufficient so as to change the binding configuration. For urea, Figure 4 shows a more drastic effect on PE complexation: 10 and 30 wt % urea moves the PE chains in the complex to a larger separation with the *g*(*r*) peaks shifting to 0.95 nm and beyond 1 nm, respectively. Careful inspection of the snapshots, supported by the density maps of Figure 2, reveals that this arises from the condensation of urea around both PEs, which may provide a more persistent solvation shell than pure water, leading to the increased backbone separation shown in the *g*(*r*) data.

**Figure 5:**
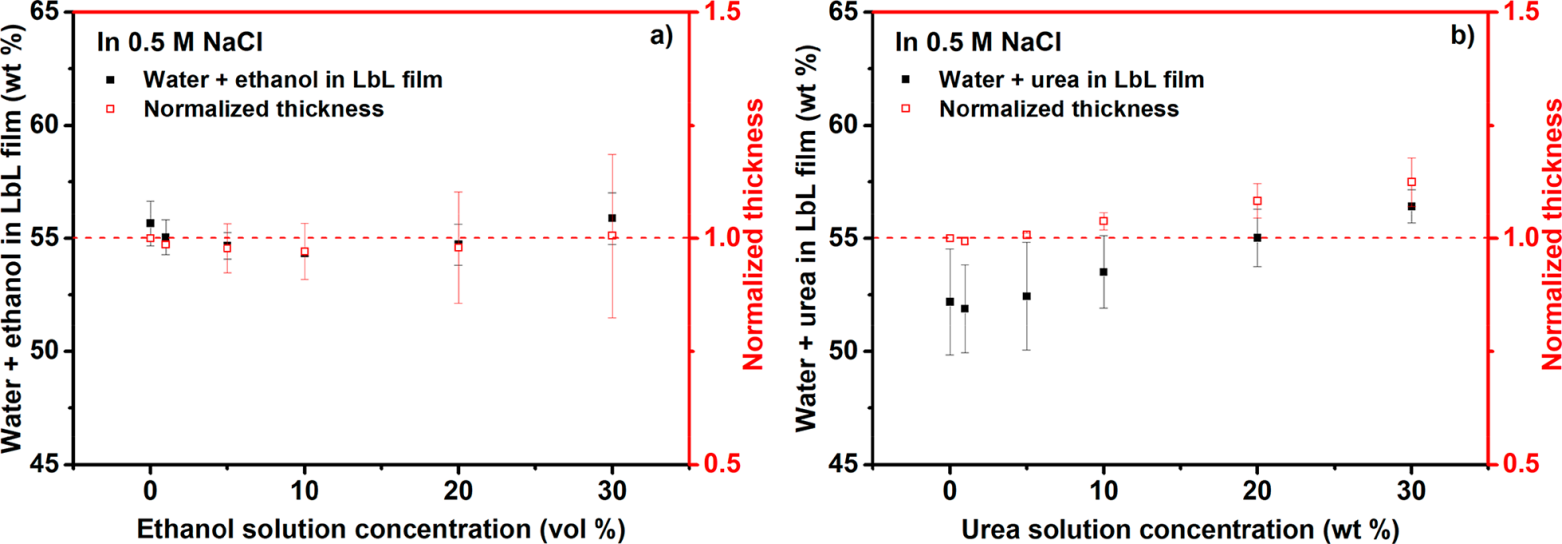
Left axis: solvent (water + additive) content of PDADMA/PSS layer-by-layer (LbL) films. Right axis: LbL film thickness normalized to the initial hydrated film thickness before exposure to varying solution concentrations (0–30 wt %) of (a) ethanol and (b) urea obtained from quartz crystal microbalance with dissipation (QCM-D) experiments.

The effects of ethanol and urea on PDADMA/PSS layer-by-layer thin film swelling were also studied experimentally by using quartz crystal microbalance with dissipation (QCM-D). Figure S9 represents typical changes in frequency and dissipation. Five layer pairs of PDADMA/PSS films were layer-by-layer (LbL) deposited onto a QCM-D crystal before flowing through solutions with increasing ethanol or urea concentration ranging from 0 to 30 wt %. Both LbL assembly and additive exposure were performed in the presence of 0.5 M NaCl. Figure 5a shows the combined weight percentage of water + ethanol as calculated from the difference between the hydrated film thickness and the dry film thickness obtained from profilometry. Upon first contact with a 1% ethanol solution, the solvent (water + ethanol) content in the LbL film slightly decreased from 55.7 to 55.1 wt %. As the concentration of ethanol in the contacting solution increased up to 10 wt %, the LbL film further contracted by the release of solvent (water + ethanol) until the content reached 54.3 wt %. Beyond 10 wt % ethanol, the solvent content in the LbL film then increased up to 55.9 wt % at 30 wt % ethanol. The normalized thickness is also displayed in Figure 5 to show the general trend. Figure S10 presents the unnormalized change in thickness. Taken together, this result reinforces the results shown in Figure 2, in which ethanol drives water out of the polyelectrolytes’ hydration shells. Consistent with these findings, a prior study of PDADMA/PSS multilayers reported a small ethanol uptake.^27^ Separately, swelling and spectroscopic studies of branched polyethylene imine/poly(acrylic acid) LbL assemblies observed contraction in ethanol and several other solvents, for which the swelling behavior strongly correlated to the solvent’s hydrogen-bonding ability.^60^

Similar to Figure 5a, Figure 5b shows the combined weight percentage of water + urea and the normalized thickness of the LbL films. Very low concentrations of urea (1–5 wt %) caused little to no change in the film thickness and solvent (water + urea) content. However, at 10 wt % urea, the LbL films began to swell, and the solvent content increased to 56.4 wt % at 30 wt % urea. The finding mirrors the simulation binding configurations (Figure 3), where the 10 and 30 wt % urea solutions increased the PE chains’ separation.

In pure water, where also the closer backbone distance binding conformation is present, the complex fluctuates between the two conformations during the simulation indicating the presence of two binding configurations that are relatively close in free energy. The presented simulation data are an average of the examined initial configurations in which the PE chains’ charge group orientations are rotated with each other. Figure S11 presents the unaveraged data sets and their time evolution. The precise distributions vary between simulation runs; i.e., the modeling does not have sufficient statistical sampling to assess the weights of the distribution peaks accurately. However, the qualitative division—i.e., pure water promoting the dual peak in binding while the system showing only the longer backbone binding configuration in the simulations with the solvent additives—persists.

At the macroscopic level, relative permittivity is a good measure for electrostatic screening in a solution. For pure water, the room temperature relative permittivity is 80.2.^61^ The relative permittivities of 10 wt % ethanol, 30 wt % ethanol, 10 wt % urea, and 30 wt % urea solutions are 73.89, 64.45, 83.14, and 90.97, respectively, estimated based on data in refs (62 and 63). As shown here, the trends in the relative permittivities fail to capture the striking changes caused by ethanol and urea; instead, a microscopic explanation is required. This explanation rises from the molecular-level changes in solvation, overriding the continuum-level charge screening by the solvent. Consequently, mean-field level theoretical descriptions of charged polymer interactions in solvent, such as the Poisson–Boltzmann (PB) model, may have challenges in capturing the solvent composition-induced changes in interactions. Mean-field PB approaches have, however, been successfully modified to better capture chemistry-specific dependencies in PE interactions in terms of ion and PE specificity.^53,64^ At longer separation distances, the electrostatic screening, i.e., the relative permittivity, naturally would dominate.

Cumulative RDF and contact data calculated between the PSS S atoms and the Na^+^ ions in the system are shown in Figures S5, S12, and S13 for the individual PEs and complexes. The PEs exhibit significant differences in ion condensation in the presence of the solvent additives. The most interesting difference occurs upon complexation of PSS with PDADMA. For PSS complexation with PDADMA in water, the complexation has a rather minor influence on Na^+^ ion condensation at the PSS chain. However, ion condensation is reduced significantly for complexes in ethanol. This is because the presence of PDADMA compensates for part of the PSS charge. Thus, the effect of ethanol condensation at the hydrophobic side of the PSS chain on the enhancement of ion condensation at the charge groups is much less than when the charged groups remain freely solvated.

**Figure 6:**
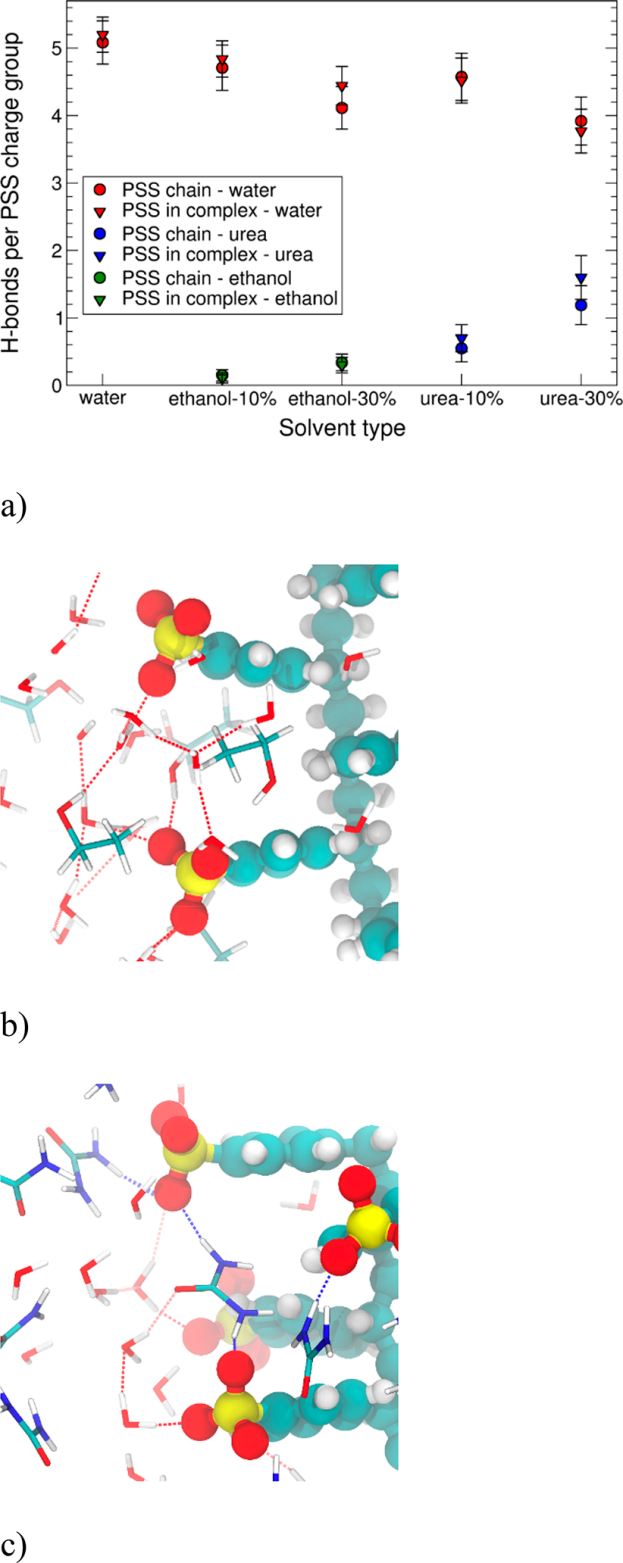
(a) Calculated average number of hydrogen bonds between PSS and water, ethanol, or urea molecules for the PE single chains (circles) and complexes (triangles). The visualizations highlight example hydrogen-bonding configurations formed in (b) 30 wt % ethanol and (c) 30 wt % urea.

To understand the nature of the observed solvation changes of the PEs and the resulting complexation changes, it is instructive to consider hydrogen bonding of PSS with water and the additives. PDADMA does not form hydrogen bonds, so it is omitted from the analysis. Figure 6 presents the number of hydrogen bonds formed per PSS charge group in the different solvent compositions for both the individual PSS chains and their complexes with PDADMA. As expected, in water, PSS forms the highest number of hydrogen bonds with the solvent. Introducing ethanol, which is a larger solvent species and is capable of forming just one hydrogen bond, significantly decreases the hydrogen bonding. Notably, the decrease in the number of PSS–water hydrogen bonds in 10 and 30 wt % ethanol systems appears to arise rather from ethanol pushing water away, i.e., the volume exclusion effect, instead of competitive hydrogen bonding between PSS and ethanol. On the other hand, in systems with urea, the total number of hydrogen bonds hardly decreases from that of pure water solvent. These quantitative observations are visualized by the snapshots in Figure 6, which present representative hydrogen-bonding configurations in 30 wt % urea and ethanol.

For small ethanol concentrations, a small decrease in PEM swelling was observed relative to the case of no ethanol. This is consistent with a weaker hydrogen bond network. On the other hand, urea caused swelling at elevated concentrations. For swelling and consecutive permeability increase, significant evidence in the literature exists.^21,22,27^ For example, in refs (21 and 22) the net-like structure of a weak PE gel was reported to be loosened by urea presumably by destabilization of the hydrogen-bonding network, consistent with our findings of urea promoting loosening via destabilization of the binding conformation. The hydrogen-bonding analysis of Figure 6, however, shows that the number of hydrogen bonds formed by PSS in water–urea solutions remains similar to pure water, indicating that the loosening in refs (21 and 22) is due to urea carrying a weaker hydrogen bond network. Furthermore, the results on urea condensation around the PE charged groups have significance in ion-complexation and ion-sensing applications as urea derivatives are used in these (see e.g. refs (65−67)). In regards to ethanol as an additive, it has been previously shown that, e.g., PSS- and PAH-based encapsulation of both urease^26^ and dextran^68^ can be controlled by small amounts of ethanol. Ethanol leads to an increase in urease or dextran diffusion through the complexed PE capsule material which indicates that the PE–PE complexation becomes looser. Ethanol has also been used to control self-healing and actuating adhesives of poly(ethylenimine)/poly(acrylic acid) (BPEI/PAA) PE LbL assemblies.^69^ The findings connect with the viscosity response observed in this work and indicate that solvent additives provide a local solvation environment (chemical group dependent) means to tuning viscoelasticity properties of PE materials.

The PE–PE ion pairs and the hydrogen bond network with the solvent carry mechanical load—we observed here that urea solvates readily both the hydrophobic and charged moieties, disrupting the hydrogen bond network and inducing swelling. The weakening of the hydrogen bond network should also manifest as a decrease in stiffness. For ethanol, the effect is two-faced as it solvates the hydrophobic backbone of PSS but depletes from the charged groups. This means ion pairing and water at ion pairs remain less influenced by the additive. However, the solvent composition variation around the PEs induces different ion solvation environments for e.g. salt ions: We expect this to lead to PE complexes in the presence of ethanol showing enhanced salt sensitivity.

It is worth noting that the effects of solvent also rise from changes to the entropy of the system, especially in the case of binary solvent where one of the components is water. The additional solvent component changes the hydrogen-bonding network of water which can have a significant change in entropy contribution of the complexation. Our simulation results show that while ethanol decreases the hydrogen-bonding significantly, urea replaces the hydrogen-bonding network loss in water by its own hydrogen bonding that has different entropic character—it is likely that the change contributes to the swelling response.

Altogether, changes in the bonding configurations and hydrogen bonding within the solvation layer can be expected to translate directly to chain diffusion characteristics and intramolecular binding in the PE materials even in the absence of direct swelling response. This means that changes in the solvent composition provide a likely means to control chain diffusion dynamics, binding in PE assemblies, and more generally the viscoelastic response. Naturally, if changing the solvent composition induces a change in solvent within the PE material, this effect will dominate (see e.g. ref (70) for the effect of water). For example, if the PE assemblies are used as a host matrix for e.g. therapeutic species, the binding of these and release have been reported to depend on hydrogen bonding in the polymer matrix.^71^ Furthermore, solution addition of urea makes the swelling response of weak PE gels under pH changes stronger.^21,22^ These observations may be explained by changes in the PE’s charge state which increases with urea condensation (see Figure 2).

## Conclusions

Molecular dynamics simulations of the effect of ethanol and urea as solvent additives to PSS–PDADMA complex formation were performed. Altogether, the simulations point toward both ethanol and urea having a significant effect on the PE material via influencing the local solvation environment which then affects PE–PE binding configurations at the microscopic level. As solvent additives, ethanol and urea have opposite effects: ethanol is depleted from around the PEs and urea condensate and replaces water as the solvent, which influences binding configurations and also counterion condensation. Zeta-potential measurements showed consistent response of PE chains in the presence of ethanol and urea solvent additives. This difference also manifested in the QCM-D characterization in which ethanol addition did not swell the PEM significantly, but urea did.

Specifically, for urea, we found that it condensates strongly around both PEs replacing water partially as the first solvation shell. In total, urea as solvent additive decreases solvation, as measured by urea’s hydrogen bonding with the solvent. Urea also leads to significant increases in PE–PE separation in the complex which corresponds to weakening of the electrostatic binding.

For ethanol, we conclude that the key to PEM materials scale response is the asymmetric solvation environment, i.e., poor solvation of the ionic groups by ethanol which leads to varying solvent microenvironment around the PEs and consequently enhanced ion condensation. In prior work,^23^ this decrease in solvent quality, i.e., increasing the amount of ethanol in assembly solution, has been reported to lead to both film thickness and mass increase.^23,25^ Here, our QCMD characterization indicated that ethanol addition leads to a decrease of solvent (water + ethanol) in the PEM for PEMs of PSS and PDADMA. The observations indicate that ethanol, because of its polar and apolar ends, is a solvent additive that enables tuning the response by PE chemistry due to the asymmetric solvation character.

In total, the effect of solvent on PE assemblies rises from the solvent affecting the energetics of the PE association. For macroscopic-scale materials, this occurs via the relative permittivity, i.e., via influencing binding enthalpy through electrostatic screening (electrostatic contributions). Our work here shows that the continuum scale relative permittivity description is insufficient to capture the response for PEs. This is because microscopic, molecular level interactions, i.e., localized ion pairing and counterion release entropy, give rise to complexation and the materials characterization. In agreement, ref (68) concludes that the change in PSS–PAH LbL capsule permeability upon ethanol or acetone addition to solvent does not connect with the electrostatic screening by the solvent but instead structural and softening changes. Altogether, the finding rises from the strong influence of PE–PE ion pairs, their local solvation, and the importance of extrinsic vs intrinsic charge compensation on the PE materials properties. These all are interactions that rise crucially from the microstructural level, thus making the microlevel correlations the dictating factor instead of the continuum materials scale electrostatic screening.

## Supporting Information Available

^1^H NMR spectra of PSS in D_2_O and the corresponding peak integrals, MDSC reversible heat flow for dry PSS, SEC chromatograms of PSS, additional radial distribution function, density profile, binding configurations, and contact number based analysis of the molecular dynamics simulations trajectories, raw and unnormalized experimental data from QCM-D measurements, as well as photos of the samples without added urea and with 1 wt % added urea (PDF)
