# Supplementary material for: Effect of Ethanol and Urea as Solvent Additives on PSS–PDADMA Polyelectrolyte Complexation
Source: Macromolecules. 2022 Apr 15;55(8):3140–50. doi: 10.1021/acs.macromol.1c02533 (PMC9052311; doi:10.1021/acs.macromol.1c02533)
Supplement: Supplementary file 1 — ma1c02533_si_001.pdf [file ma1c02533_si_001.pdf]

Supporting Information for

Effect of ethanol and urea as solvent additives on PSS-PDADMA polyelectrolyte complexation

Mohammad Khavani[§], Piotr Batys[‖], Suvesh M. Lalwani[†], Chikaodinaka I. Eneh[†], Anna Leino[§], Jodie L. Lutkenhaus[†‡], and Maria Sammalkorpi*[§⊥⫰]*

§ Department of Chemistry and Materials Science, School of Chemical Engineering, Aalto University, P.O. Box 16100, FI-00076 Aalto, Finland

‖ Jerzy Haber Institute of Catalysis and Surface Chemistry, Polish Academy of Sciences, Niezapominajek 8, PL-30239 Krakow, Poland

[†]Artie McFerrin Department of Chemical Engineering and [‡]Department of Materials Science and Engineering, Texas A&M University, College Station, Texas 77843, United States

⊥ Department of Bioproducts and Biosystems, School of Chemical Engineering, Aalto University, P.O. Box 16100, FI-00076 Aalto, Finland

⫰ Academy of Finland Centre of Excellence in Life-Inspired Hybrid Materials (LIBER), Aalto University, P.O. Box 16100, FI-00076 Aalto, Finland

Supplementary materials

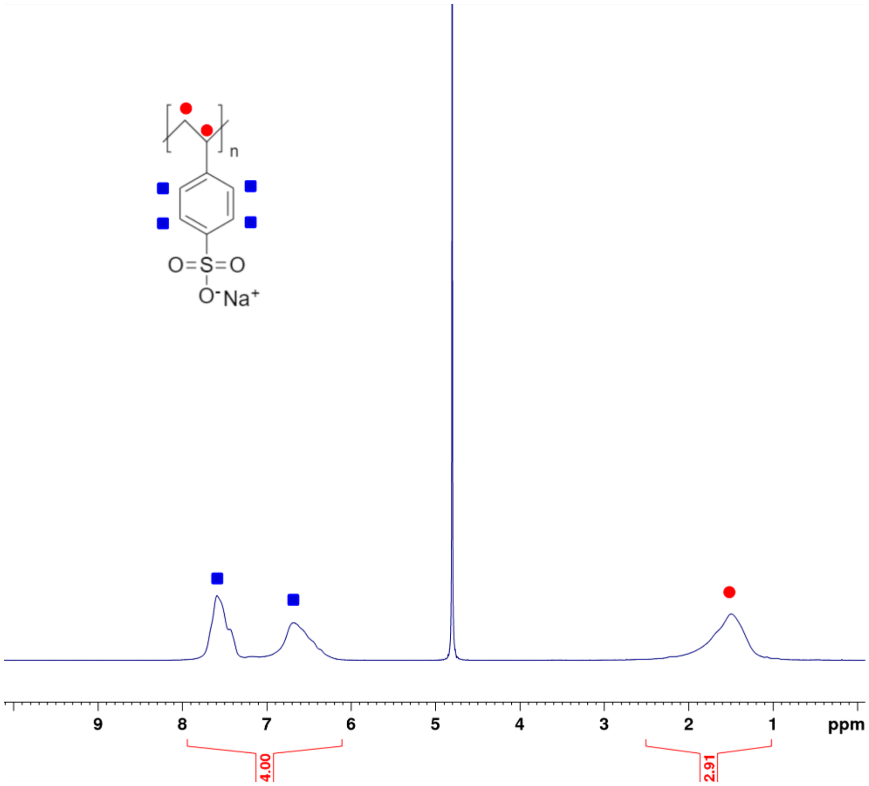

Figure S1. ^{1}H NMR spectra of PSS in D_2O and the corresponding peak integrals.

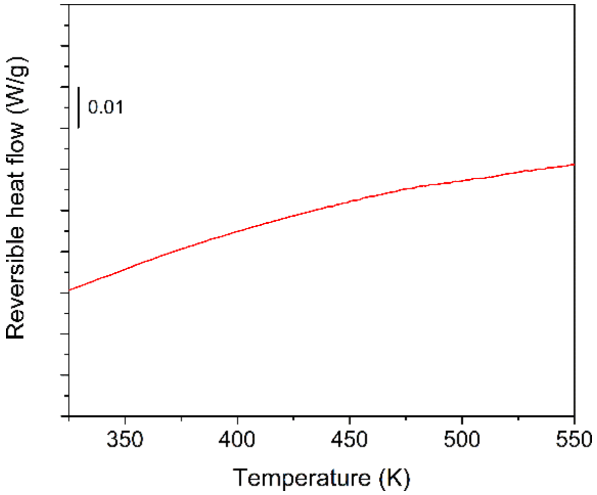

Figure S2. MDSC reversible heat flow for dry poly(sodium-4 styrene sulfonate). The thermogram is shown in “exotherm down” format. The ramp rate was 3 °C/min, and the amplitude was 0.64 °C.

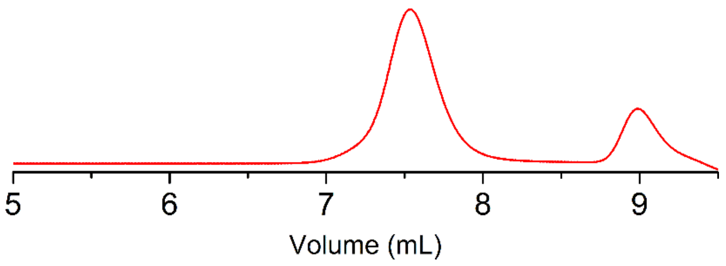

Figure S3. SEC chromatograms of PSS. Number-average molecular weight M_n = 28,234 g/mol and dispersity $Đ$ = 1.12 correspond to the first peak.

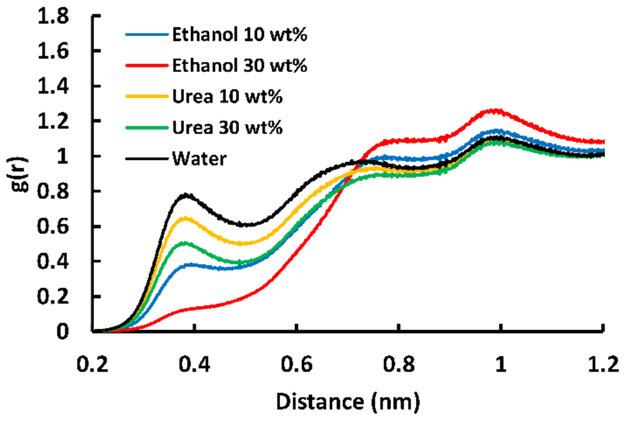

(a)

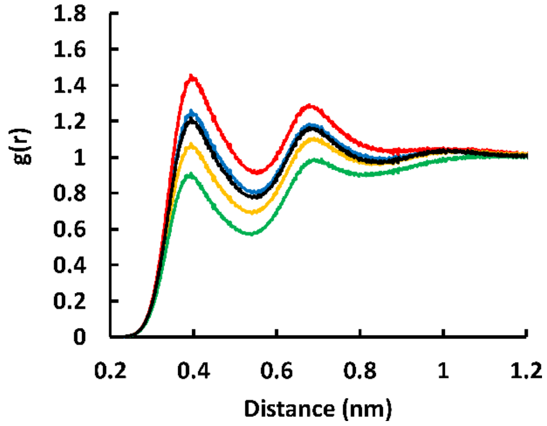

(b)

Figure S4. The calculated 2D radial distribution function *g(r)* for the backbone atoms of PSS (a) and PDADMA (b) single chains with water molecules. The legend in (a) applies to (b).

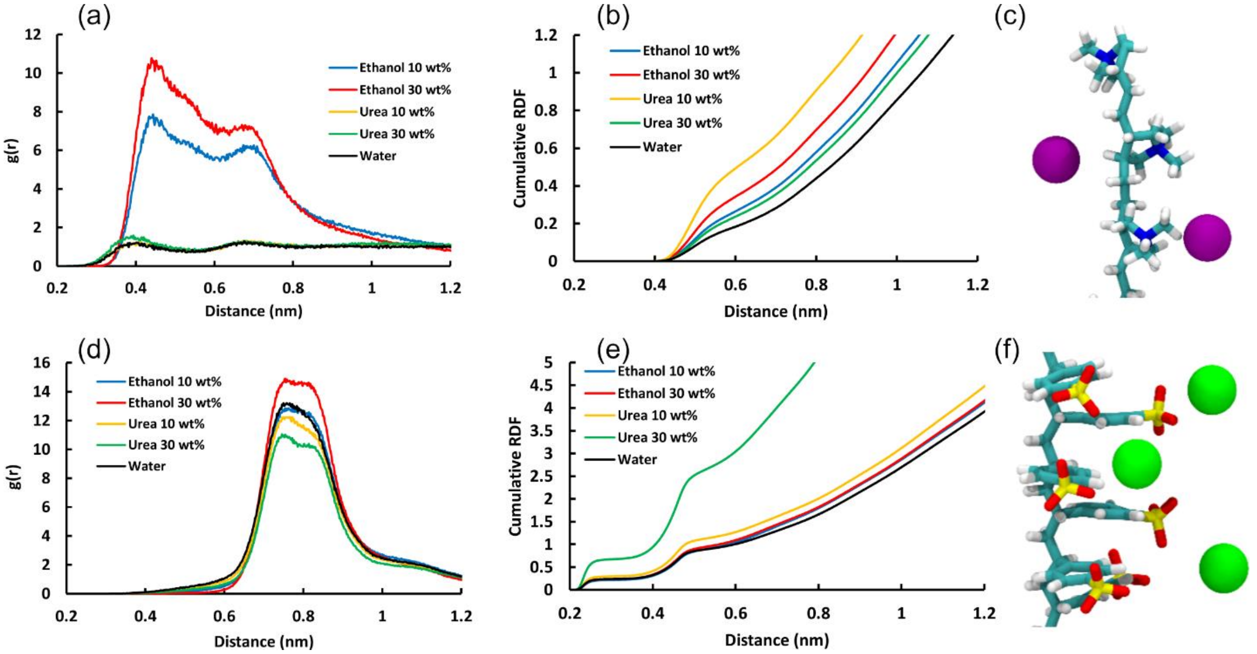

Figure S5. Effect of ethanol and urea as solvent additives on the counterion condensation around single PSS and PDADMA chains. The calculated 2D radial distribution function, *g(r)*, (a) for the backbone atoms of single chain of PDADMA and Cl^- ions, (b) the corresponding cumulative RDF, and (c) a typical chain conformation with a condensed Cl^- ion for the PDADMA system. (d and e) The corresponding data for PSS single chain and Na^+ counter ion, and (f) the corresponding sodium ion snapshot for the PSS system, respectively.

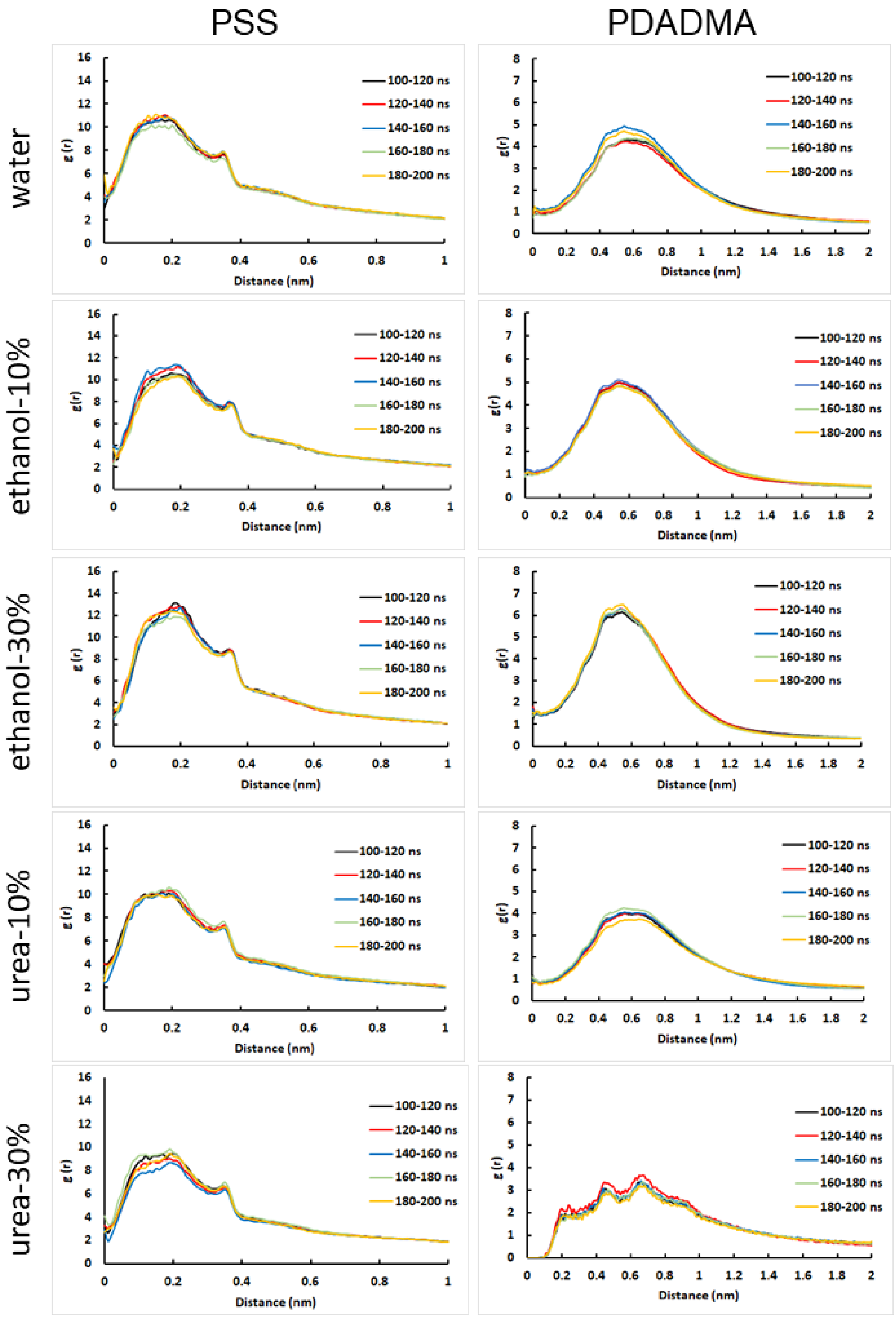

Figure S6. Time evolution of the calculated 2D radial distribution function, *g(r)*, for PSS S atoms and Na^+ ions and PDADMA N atoms and Cl^- ions. The time evolution is presented to demonstrate

that configurations have converged in time evolution over the analysis time period 100 ns – 200 ns.

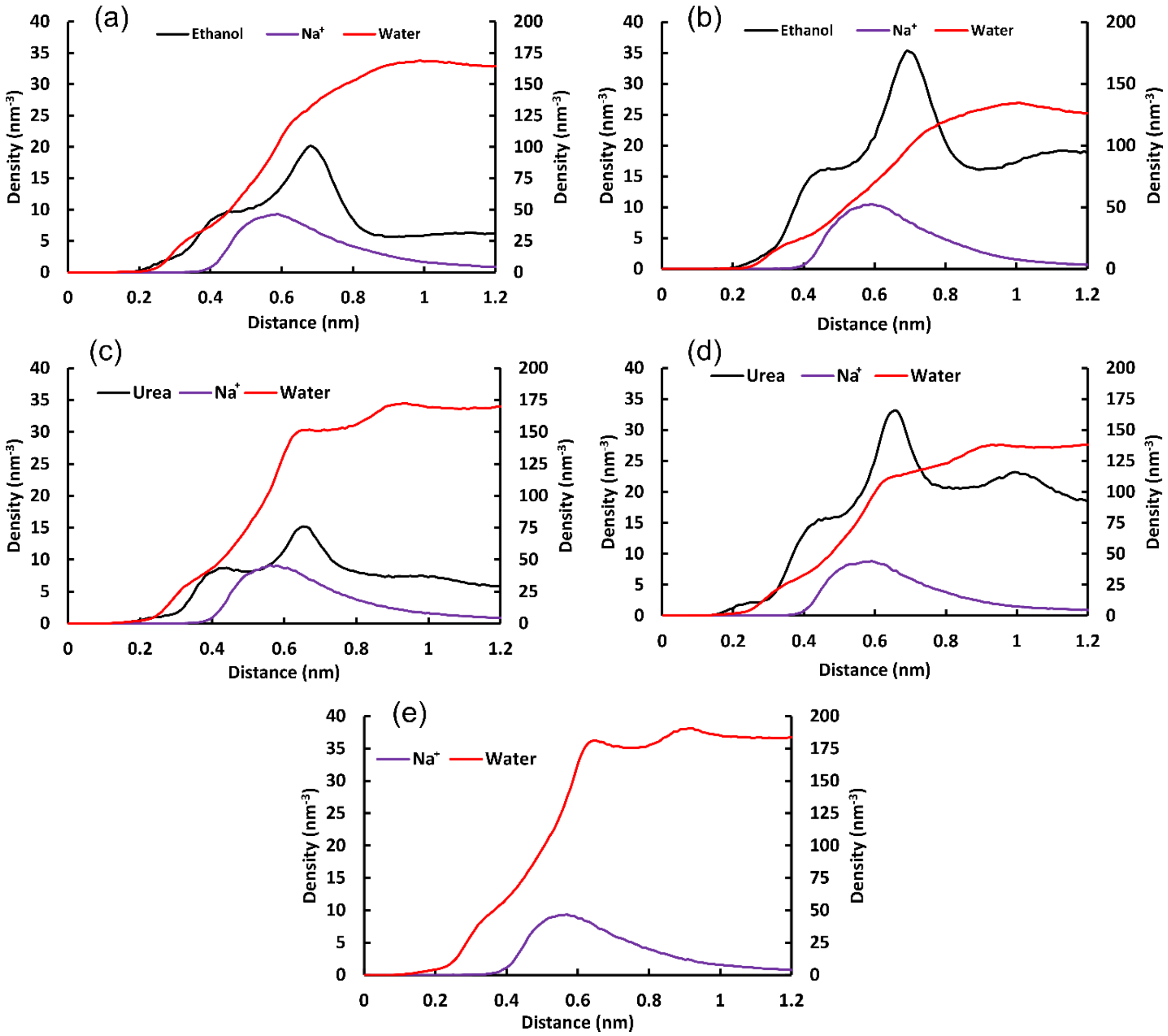

Figure S7. The solvent and ion number density calculated around the backbone atoms of PSS in water and solvent mixtures, (a) 10 wt% ethanol, (b) 30 wt% ethanol, (c) 10 wt% urea, (d) 30 wt% urea, and (e) pure water. The left and right *y* axes show the number density values for the ions and the solvent additive or water, respectively.

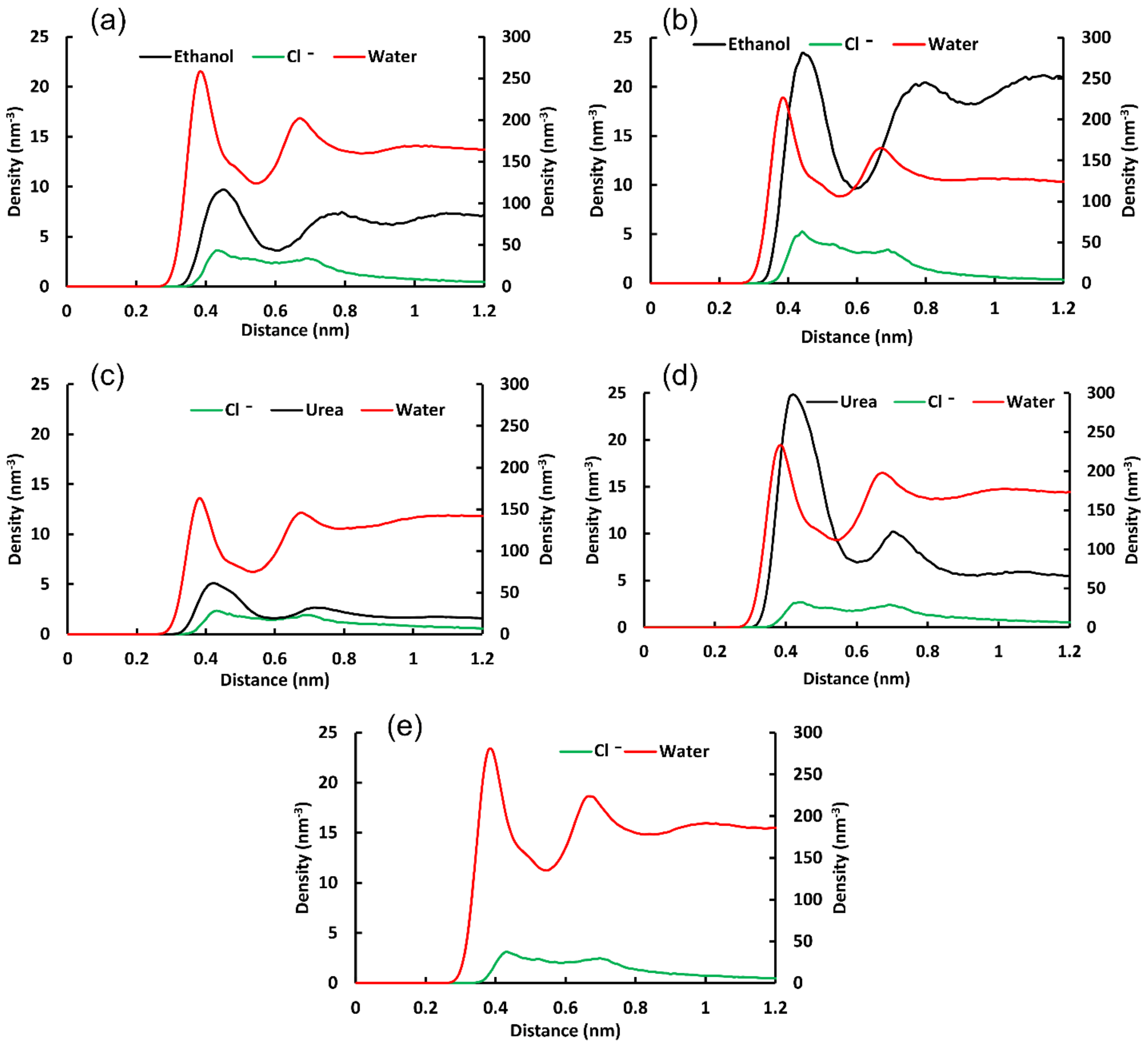

Figure S8. The solvent and ion number density calculated around the backbone atoms of PDADMA in water and solvent mixtures, (a) 10 wt% ethanol, (b) 30 wt% ethanol, (c) 10 wt% urea, (d) 30 wt% urea, and (e) pure water. The left and right *y* axes show the number density values for the ions and the solvent additive or water, respectively.

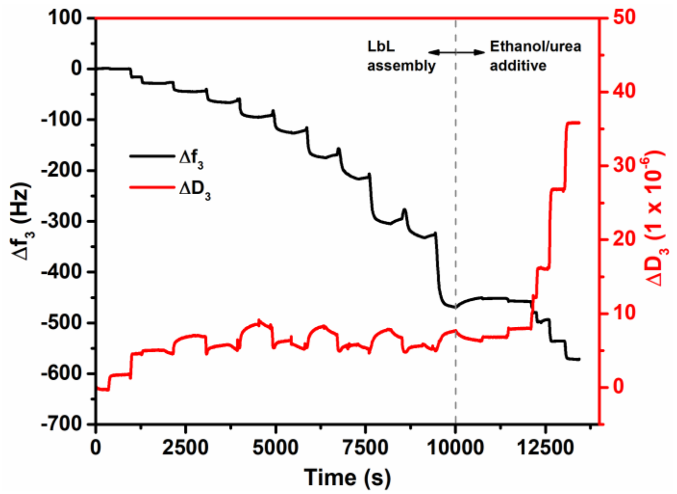

Figure S9. Representative raw experimental data from QCM-D monitoring. Each deposition step was confirmed with a step change in frequency and dissipation. A decrease in frequency is ascribed to an increase in thickness and mass whereas an increase in dissipation corresponds to an increase in the viscous component of the film.

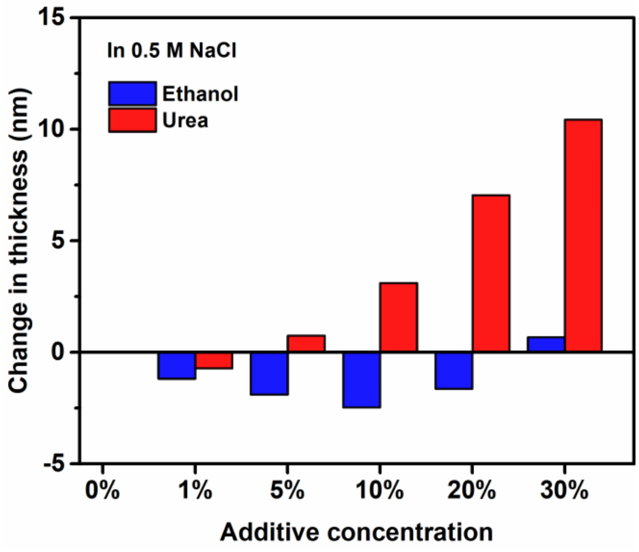

Figure S10. Changes in thickness relative to thickness at 0 wt% additive solution concentration (i.e., pure water).

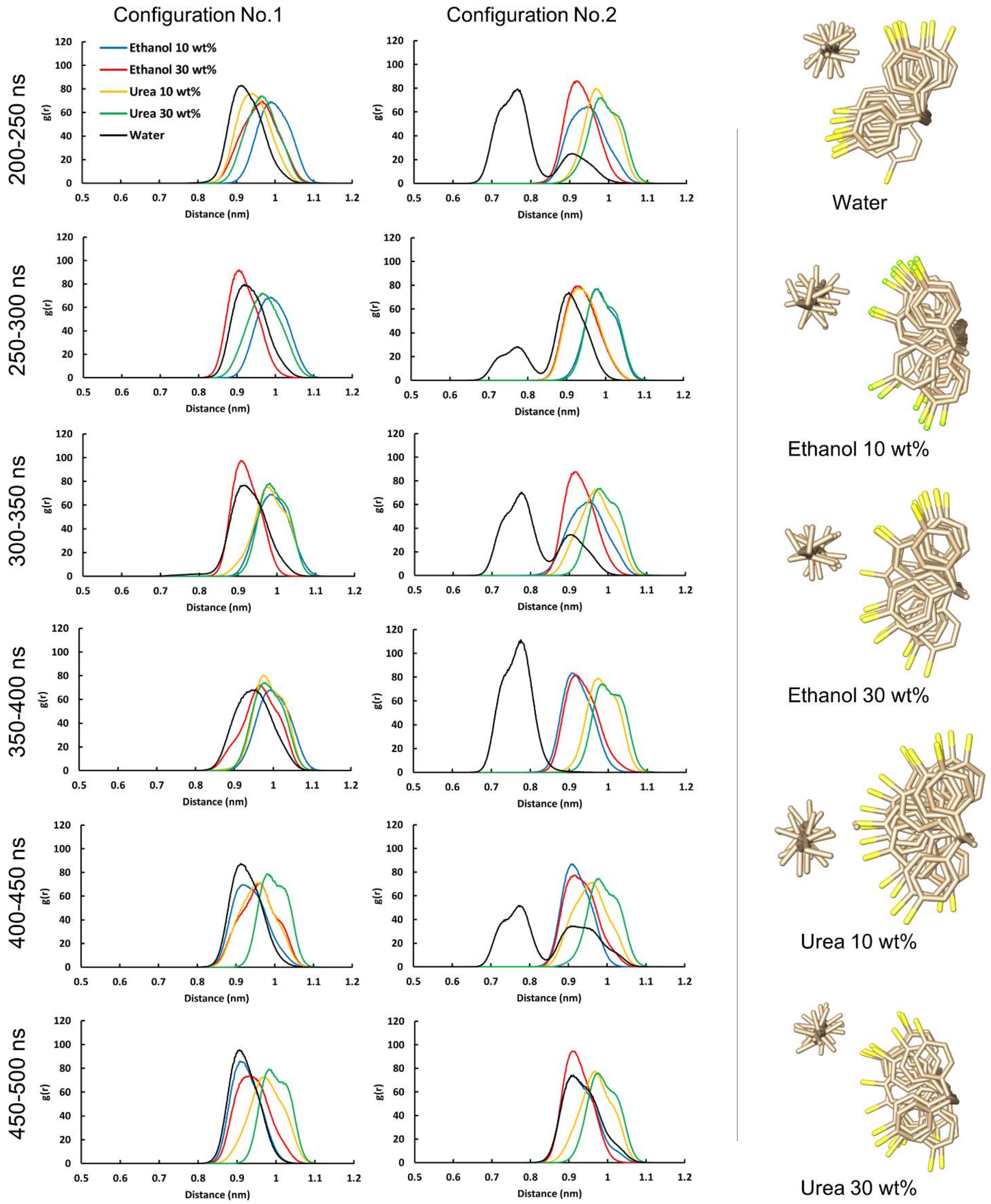

Figure S11. 2D radial distribution functions, *g(r)*, calculated between the backbone atoms of each PE for the initial configuration 1 and 2 at 50 ns time intervals of the simulation run between 200 ns and 500 ns. At right, the obtained polyelectrolyte configurations are presented. Initial 200 ns were disregarded in the analysis as complex formation and relaxation time.

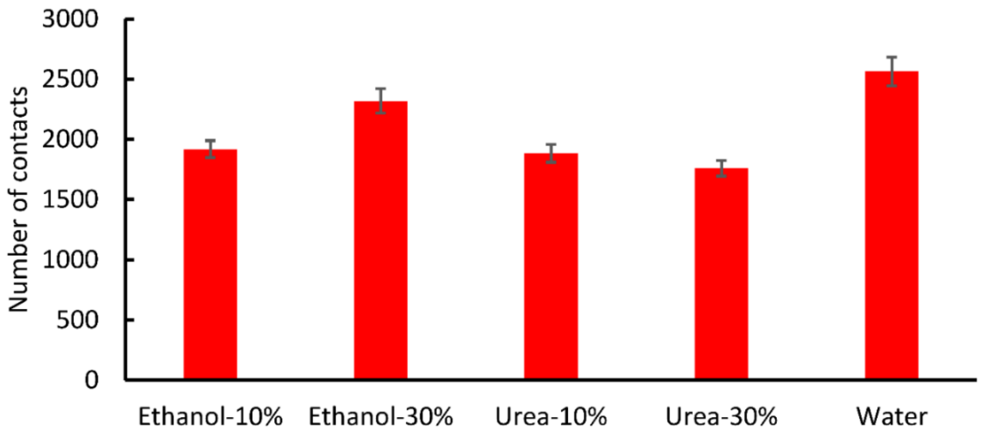

(a)

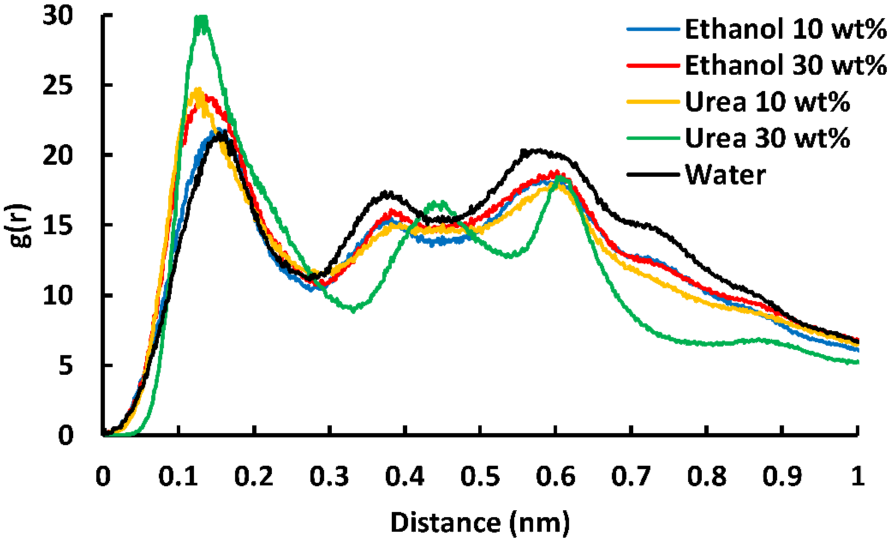

(b)

Figure S12. Additional complex structural characterization. (a) Mean number of contacts between the PSS and PDADMA chains in the complexes and (b) the radial distribution function *g(r)* plots calculated between the PE charge group centers (S atom in PSS and N atom in PDADMA). The analysis is done for the 200-500 ns time period of the MD simulations.

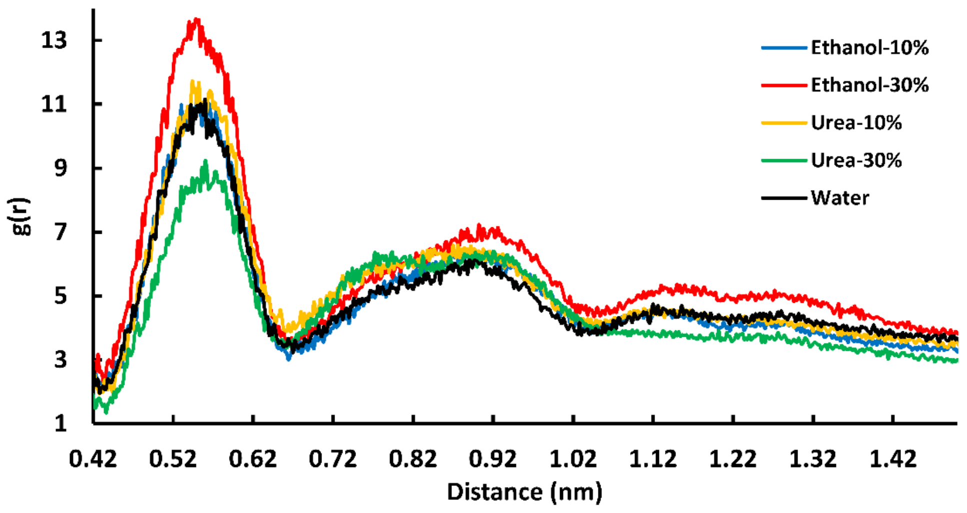

(a)

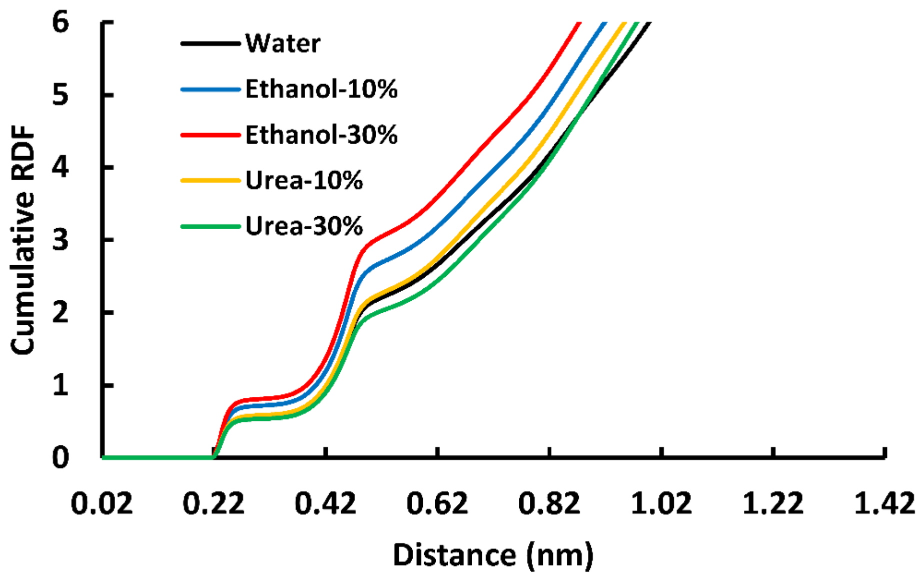

(b)

Figure S13. (a) The calculated radial distribution function *g(r)* and (b) the cumulative radial distribution function for the S atoms of PSS and Na^+ ion in PSS-PDADMA complexes.

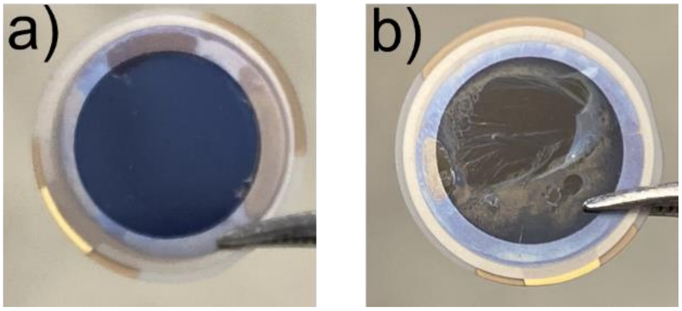

Figure S14. An LbL-coated QCM-D crystal (a) in an experiment without urea and (b) after addition of 1 wt% urea, in which visible film detachment is shown. Similar observations were made also at higher concentrations of ethanol (10 wt%).
